# Interleukin-22 attenuates allergic airway inflammation in ovalbumin-induced asthma mouse model

**DOI:** 10.1186/s12890-021-01698-x

**Published:** 2021-11-26

**Authors:** Jingru Wang, Shengnan Gao, Jingyuan Zhang, Chunxiao Li, Hongwen Li, Jiangtao Lin

**Affiliations:** 1 Peking University China-Japan Friendship School of Clinical Medicine, No. 2, East Yinghua Road, Chaoyang Disteict, Beijing, 100029 China; 2 Department of Pulmonary and Critical Care Medicine, China-Japan Friendship Hospital, Beijing, 100029 China; 3 Department of Pulmonary and Critical Care Medicine, Graduate School of Chinese Academy of Medical Sciences, Peking Union Medical College, China-Japan Friendship Hospital, Beijing, 100730 China

**Keywords:** Allergic asthma, Cytokines, Interleukin-22, Animal model, Ovalbumin sensitization

## Abstract

**Background:**

Allergic asthma is a chronic airway inflammatory disease with a number of cytokines participating in its pathogenesis and progress. Interleukin (IL)-22, which is derived from lymphocytes, acts on epithelial cells and play a role in the chronic airway inflammation. However, the actual role of IL-22 in allergic asthma is still unclear. Therefore, we explored the effect of IL-22 on allergic airway inflammation and airway hyperresponsiveness (AHR) in an ovalbumin (OVA)-induced asthma mouse model.

**Methods:**

To evaluate the effect of IL-22 in an allergic asthma model, BALB/c mice were sensitized and challenged with OVA; then the recombinant mouse IL-22 was administered intranasally 24 h prior to each challenge. The IL-22 levels in lung homogenates and bronchoalveolar lavage fluid (BALF) were measured by enzyme linked immunosorbent assay, respectively. AHR was evaluated through indicators including airways resistance (Rrs), elastance (Ers) and compliance (Crs); the inflammatory cell infiltration was assessed by quantification of differential cells counts in BALF and lung tissues stained by hematoxylin and eosin (H&E); IL-22 specific receptors were determined by immunohistochemistry staining.

**Results:**

The concentration of IL-22 was significantly elevated in the OVA-induced mice compared with the control mice in lung homogenates and BALF. In the OVA-induced mouse model, IL-22 administration could significantly attenuate AHR, including Rrs, Ers and Crs, decrease the proportion of eosinophils in BALF and reduce inflammatory cell infiltration around bronchi and their concomitant vessels, compared with the OVA-induced group. In addition, the expression of IL-22RA1 and IL-10RB in the lung tissues of OVA-induced mice was significantly increased compared with the control mice, while it was dramatically decreased after the treatment with IL-22, but not completely attenuated in the IL-22-treated mice when compared with the control mice.

**Conclusion:**

Interleukin-22 could play a protective role in an OVA-induced asthma model, by suppressing the inflammatory cell infiltration around bronchi and their concomitant vessels and airway hyperresponsiveness, which might associate with the expression of its heterodimer receptors. Thus, IL-22 administration might be an effective strategy to attenuate allergic airway inflammation.

## Background

Asthma is one of the most common chronic airway inflammatory disorders that affects 358.2 million individuals worldwide, up by 12.6% [1]. The China Pulmonary Health (CPH) study has reported that the overall prevalence of asthma was 4.2% in adults [2]. Allergic asthma is the most common subtype of asthma [3], characterized by chronic airway inflammation and hyperresponsiveness, affecting approximately 60% of all asthma patients [4]. Airway epithelial cells and immune cells are occupied in bidirectional communication to regulate the chronic airway inflammation [5]. Cytokines are involved in asthmatic chronic airway inflammation, especially those derived from the lymphocyte, which acts on epithelial cells, such as cytokine interleukin (IL)-22.

Interleukin-22 is a member of the IL-10 family, and predominately derives from innate and adaptive immune cells, including αβ T cells, γδ T cells, natural killer T cells and group 3 innate lymphoid cells (ILC3s) [6–9]. It functions as a bridge by binding to its type 2-cytokine receptor, which is composed of a heterodimer of IL-22 receptor A1 (IL-22RA1) and IL-10RB; the former is expressed on non-hematopoietic cells, especially epithelial cells, but not immune cells [10, 11], while the latter is expressed on hematopoietic and non-hematopoietic cells [11]. By combining with IL-22RA1 and IL-10RB, IL-22 activates target cells to participate in inflammation, antimicrobial immunity and tissue repair [12], possibly playing an important role in the chronic airway inflammation of asthma. IL-22-binding protein (IL-22BP), also known as IL-22RA2, is a soluble form of the IL-22 receptor homolog, which binds IL-22 with high affinity and neutralizes the activity of IL-22 in vitro [13, 14].

IL-22 plays bidirectional roles in allergic airway inflammation, both protective and pathogenic. On the one hand, the concentration of IL-22 significantly increases in peripheral blood mononuclear cells (PBMC), serum and lung tissues of asthma patients compared with healthy controls [15–18]. In mouse models of asthma, the IL-22 level increases in the lung after airway challenge [19]. IL-22 could induce antimicrobial protein, Reg3γ, produced by signal transducer and activator of transcription (STAT)3 activation, which then reduced the secretion of IL-33 and thymic stromal lymphopoietin (TSLP) of the lung epithelial cells, and inhibited the eosinophilic airway inflammation [20]; IL-22 could also suppress proinflammatory cytokine interferon-γ (IFN-γ)-induced secretion of proinflammatory chemokines in human bronchial epithelial cells in vitro [21], demonstrating that it may have a potential protective role in asthma. Besides, in IL-22-deficient (IL-22^−/−^) mice or administration anti-IL-22 antibody wild-type (WT) mice, allergic airway inflammation and airway hyperresponsiveness (AHR) was aggravated, manifested as an enhanced proportion of eosinophils in bronchoalveolar lavage fluid (BALF), exacerbated inflammatory cell infiltration around the bronchi and their concomitant vessels, increased airway responsiveness and cytokine production [9, 19, 20], indicating the protective role of IL-22 in allergic airway inflammation. On the other hand, IL-22 deficiency or administration of anti-IL-22 antibody could dramatically decrease the proinflammatory cytokine production, eosinophil recruitment, AHR and mucus secretion [22–24], suggesting IL-22 may play a pathogenic role in allergic airway inflammation. Therefore, the effects of IL-22 on allergic asthma airway inflammation still need further study.

In view of the nonalignment of IL-22 roles in allergic asthma airway inflammation, we set out to investigate the role of IL-22 in the pathogenesis of allergic airway inflammation in asthma mouse model.

## Methods

### Mice

Wild type female BALB/c mice (aged 6–8 weeks) were purchased from Vital River Laboratory (License No. SCXK (Jing) 2019-0009, Beijing, China). All BALB/c mice were randomly divided into different groups (10 mice per group), and were maintained in standard animal housing conditions (12 h light and 12 h dark cycles, and freely obtaining food and water) of Animal Laboratory of China-Japan Friendship Hospital. All protocols on mice were approved by the Animal Ethical and Welfare Committee of China-Japan Friendship Hospital (approval No. zryhyy21-20-01-3).

### Ovalbumin (OVA)-induced allergic airway inflammation

**Fig. 1:**
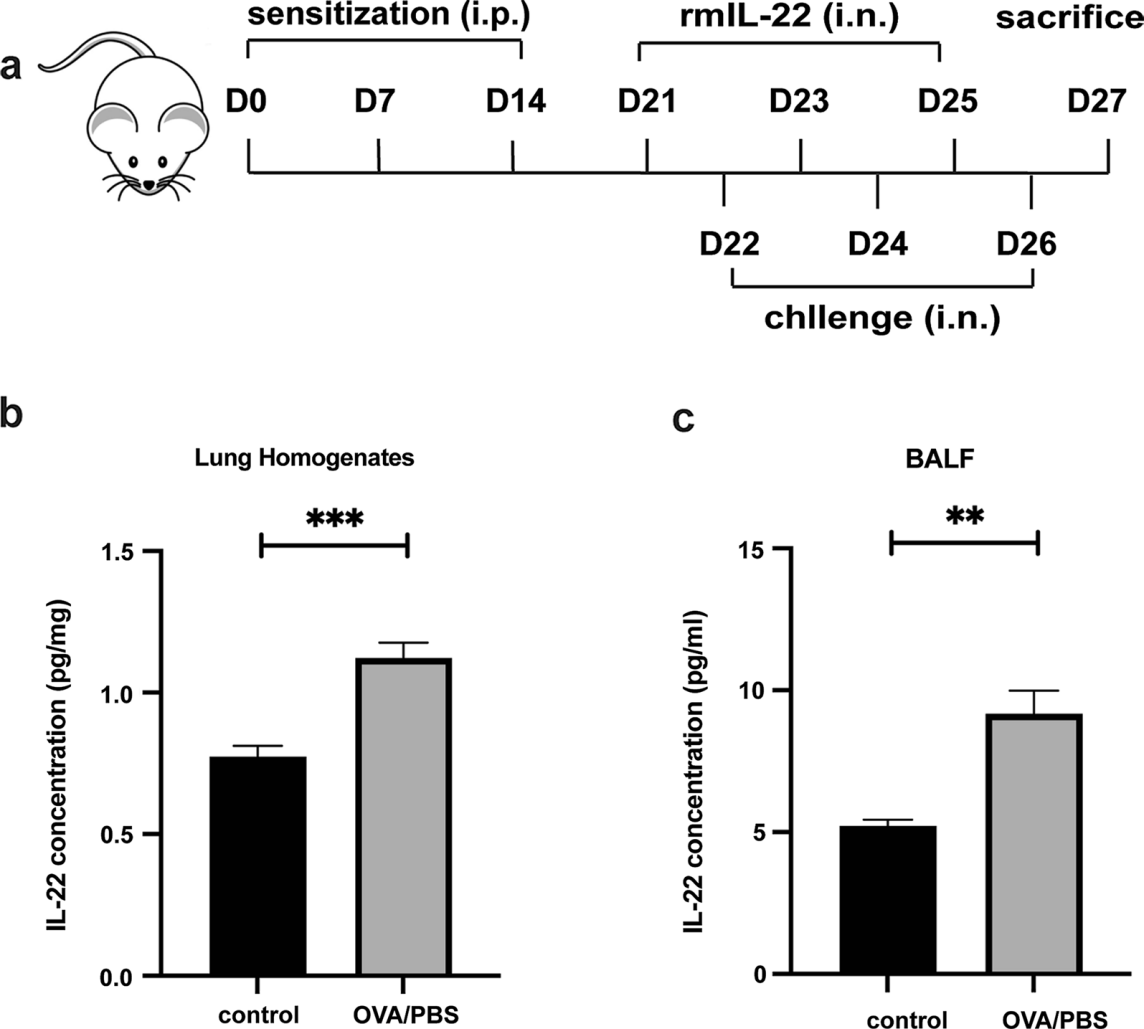
The schedule for the OVA-induced asthma model and the expression of IL-22 in OVA-induced asthma model. **a** Schedule for the OVA-induced asthma model; **b** concentration of IL-22 in lung homogenates (n = 8); **c** concentration of IL-22 in BALF(n = 4–8). Bars are presented as the mean ± SEM. i.n. intranasal, i.p. intraperitoneal; * *p* < 0.05, ** *p* < 0.01, *** *p* < 0.001

The OVA-induced mouse model of asthma was established as described previously [25, 26]. BALB/c mice were divided into three groups: the phosphate buffered saline (PBS) control group (control), ovalbumin group (OVA/PBS), and OVA + recombinant IL-22 group (OVA/IL-22). BALB/c mice were anesthetized with isofluorane inhalation before the intranasal administration. At Days 0, 7, and 14, mice were sensitized by intraperitoneal injection with 100 µg OVA (Sigma-Aldrich, St. Louis, MO, USA; grade V) and 2.25 mg aluminum hydroxide in PBS or equivalent PBS (200 µl per mouse); then they were challenged by inhaled OVA (100 µg in 50 µl PBS per mouse) or equivalent PBS on Days 22, 24, and 26. The recombinant mouse IL-22 (rmIL-22, BD Biosciences, San Diego, Calif, USA) (0.2 µg per mouse) (as the OVA/IL-22 group) or PBS (as the control and OVA/PBS group) was administered intranasally 24 h before the inhaled OVA challenge. As described previously with minor modifications [19, 22, 24, 27], we have chosen the dose of rmIL-22 (0.2 µg per mouse). The BALB/c mice were sacrificed 24 h after the last OVA challenge (Fig. 1a).

### Assessment of airway responsiveness

Twenty-four hours after the last intranasal OVA challenge, airway responsiveness was assessed on day 27 using the FlexiVent system (SCIREQ, Inc., Montreal, Canada) following the manufacturer's protocol, as previously described [28]. Briefly, after the anesthetization with 80 mg/kg pentobarbital sodium (Sigma-Aldrich, St. Louis, MO, USA) by intraperitoneal injection, mice were conducted with tracheotomy and endotracheal intubation. And then airway responsiveness was assessed by measuring the changes of lung resistance after sequentially exposed to increasing doses of methacholine (Mch, Sigma-Aldrich, St. Louis, MO, USA) in sterile saline (0, 6.25, 12.5, 25 and 50 mg/ml). The values of airways resistance (Rrs), elastance (Ers) and compliance (Crs) were recorded for analyzing the AHR.

### Collection of BALF and quantification of differential cells counts

After the measurement of airway responsiveness, BALF was collected by instilling and retrieving 500 µl of cold sterile saline into the lung tissues, and this above process was repeated three times [29]. And then BALF was centrifuged at 1500 g for 10 min at 4 °C, supernatants were stored at − 80 °C for further use, whereas the cell pellets were resuspended in 50 µl of sterile PBS to calculate differential cell counts (eosinophils, neutrophils, lymphocytes and macrophages) by Wright-Giemsa (BaSO, Zhuhai City, Guangdong, China) staining. And 400 cells were counted in each section in continuous fields of vision.

### Lung histopathology

Following the collection of BALF, the mice were euthanized with pentobarbital sodium. Left lung lobes were fixed by 10% neutral-buffered formalin for 24 h, and then embedded in paraffin. Finally, paraffin-embedded sections (5 µm) were stained with haematoxylin/eosin (H&E) to assess the scores of inflammatory cells infiltration, as previously described [30]. The numbers of peribronchiolar and perivascular infiltrating inflammatory cells were scored as follows: 0, no cells; 1, a few cells; 2, a ring of inflammatory cells, one cell layer of peribronchial cells; 3, a ring of inflammatory cells, two to four cells layers of peribronchial cells; and 4, a ring of inflammatory cells, more than four cell layers of peribronchial cells. Independent operators ignorant of the origin of the sections performed. Five to ten airways were counted in per mouse.

### Lung immunohistochemistry

Immunohistochemistry was used to determine the location of IL-22 specific receptor. After heated in incubator at 60 °C for 2 h, paraffin-embedded sections were treated by immersion in xylene and gradient alcohol solutions for deparaffinization and hydration, respectively, and then by sinking in 10 mM citrate buffer for antigen recovery. Then, after the endogenous peroxidase activity was blocked by 3% H_2_O_2_, sections were administrated with 10% goat serum blocking solution to reduce the nonspecific absorption of immunoglobulin. Finally, the specimens were incubated with polyclonal antibodies, anti-IL-22RA1 antibody (1:500, Merk-Millipore, Temecula, CA, USA), anti-IL-10RB antibody (1:500, Bioss, Beijing, China), and anti-IL-22BP antibody (1:500, Abcam, Cambridge, CB2 0AX, UK) at 4 °C for overnight, and then stained with a goat anti-rabbit IgG secondary antibody-HRP (1:500, Thermo Fisher Scientific, Rockford, IL, USA) at 37 °C for 30 min, according to manufacturer's protocols. Image J software was used to evaluate protein expression. Ten digital photographs of bronchioles in each tissue section were observed under 40× magnification. Data were presented as a percentage of the positively stained area in whole segments of lung tissues.

### IL-22 expression in lung homogenates and BALF

The right lung tissues were weighed and homogenized in cold PBS containing 2% protease inhibitor and phosphatase inhibitors cocktail (Solarbio, Beijing, China) by highly speed homogenizer (IKA, Guangzhou, Guangdong, China). The lung homogenates were centrifugated at 4 °C, 14,000 g for 10 min, then the sediments were removed and the supernatants were collected. The IL-22 levels in lung homogenates and BALF were determined by enzyme linked immunosorbent assay (ELISA) Kit (MULTI SCIENCES, Hangzhou City, Zhejiang, China). The procedures were executed following the manufacturer's instructions. The absorbance was measured at 450 nm and 570/630 nm by microplate spectrophotometer (Thermo Fisher Scientific, Waltham, MA USA) and data were expressed as pg/ml (sensitivity 0.74 pg/ml).

### Statistical analysis

The data analyses and graphs preparation were performed by SPSS 20.0 (IBM, Armonk, New York, USA) and GraphPad Prism 9.0 (GraphPad, San Diego, CA, USA). When the sample size was less than 50, Kolmogorov–Smirnov was used to analyzed whether the experimental data obey the normal distribution. For normal distribution data, independent Student t test or one-way analysis of variance (ANOVA) was used for comparing different groups; Wilcoxon signed rank test and Kruskal–Wallis test were performed for non-normally distributed data. Data were presented as mean ± standard error of mean (SEM). The statistical analysis involved in this study used a two-sided test, and *p* < 0.05 was considered statistically significant.

## Results

### IL-22 expression increased in OVA-induced mouse model

BALB/c mice were sensitized and challenged by OVA, and treated per-nasally with rmIL-22 or PBS control as described in the methods (Fig. 1a). The ELISA Kit analysis of lung homogenates and BALF demonstrated that OVA-induced asthma, compared with phosphate buffered saline diluent control challenge of the BALB/c mice, was associated with significant elevation of the mean concentrations of IL-22 (Fig. 1). The mean concentration of IL-22 was significantly elevated in the OVA-induced mice compared with the control mice treated with PBS challenge in lung homogenates (Fig. 1b, 1.12 ± 0.06 pg/mg vs. 0.77 ± 0.04 pg/mg, *p* < 0.001). Again, this phenomenon similarly occurred in BALF that OVA-induced mice showed a significantly increased mean concentration of IL-22 compared with those challenged with phosphate buffered saline control (Fig. 1c, 9.18 ± 0.82 pg/ml vs. 5.23 ± 0.21 pg/ml, *p* < 0.01).

### IL-22 attenuated OVA-induced airways hyperresponsiveness

**Fig. 2:**
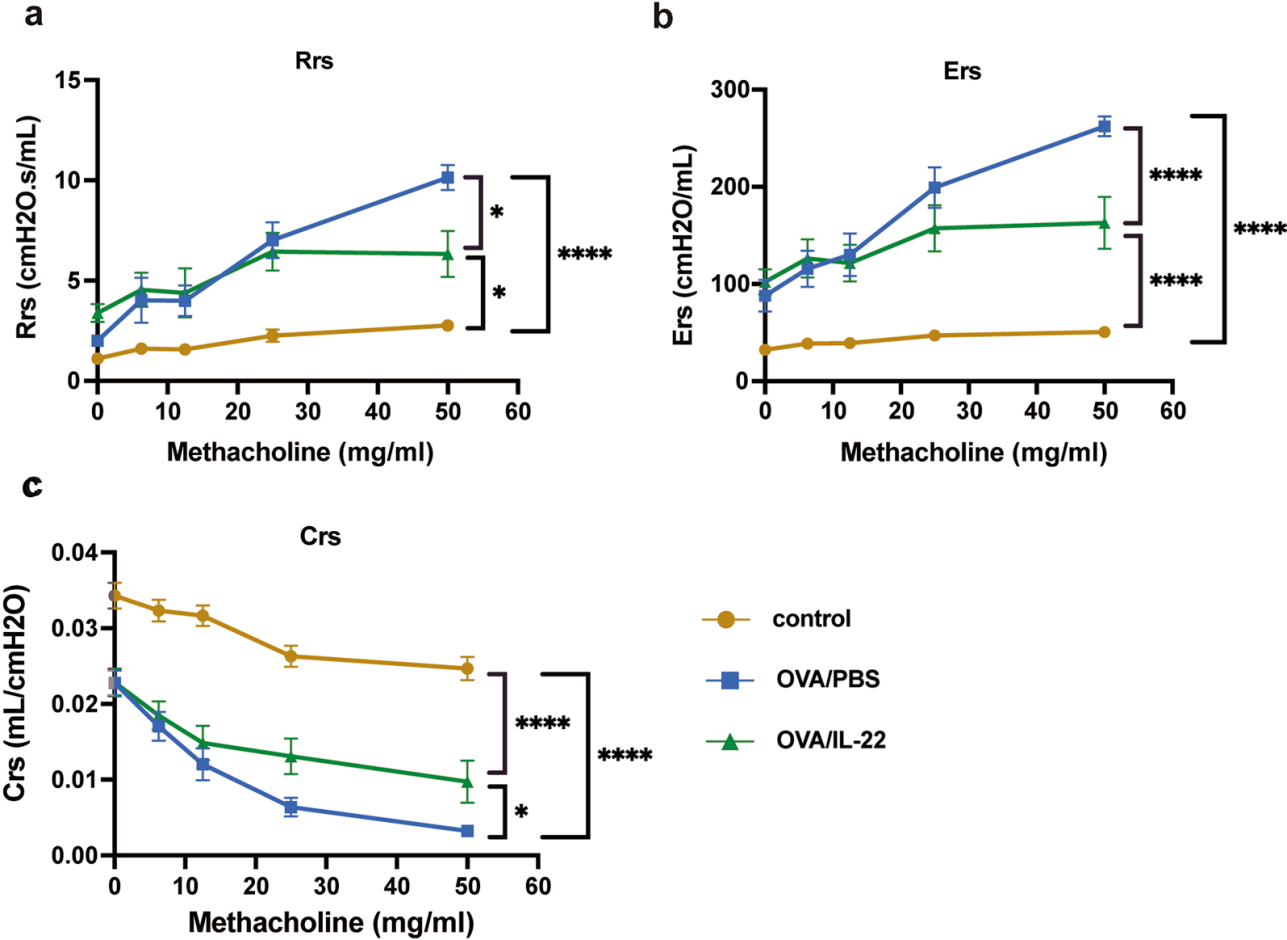
The effects of IL-22 on airway hyperresponsiveness in OVA-induced asthma model. The values of Respiratory system resistance (Rrs) (**a**), Elastance (Ers) (**b**), and Compliance (Crs) (**c**) were measured 24 h after the last OVA challenge by exposure to increasing doses of methacholine (n = 8). Bars are presented as the mean ± SEM. * *p* < 0.05, ** *p* < 0.01, *** *p* < 0.001

To determine the effect of IL-22 on airway function, we exposed BALB/c mice to increasing doses of methacholine aerosols. Administration of rmIL-22 generated a significant improvement in the methacholine-induced airways resistance compared with PBS treatment in the OVA-induced asthma model, consistent the previous report [19]. Compared with the PBS-treated control group, the Rrs (Fig. 2a) and Ers (Fig. 2b) values were significantly elevated in OVA-induced mice; meanwhile the Crs values were dramatically decreased (Fig. 2c). However, these phenomena, upregulation of Rrs and Ers and downregulation of Crs, were significantly reversed by administration with IL-22, but not completely compared with control group (Fig. 2).

### IL-22 attenuated OVA-induced inflammatory cellular infiltration in BALF

**Fig. 3:**
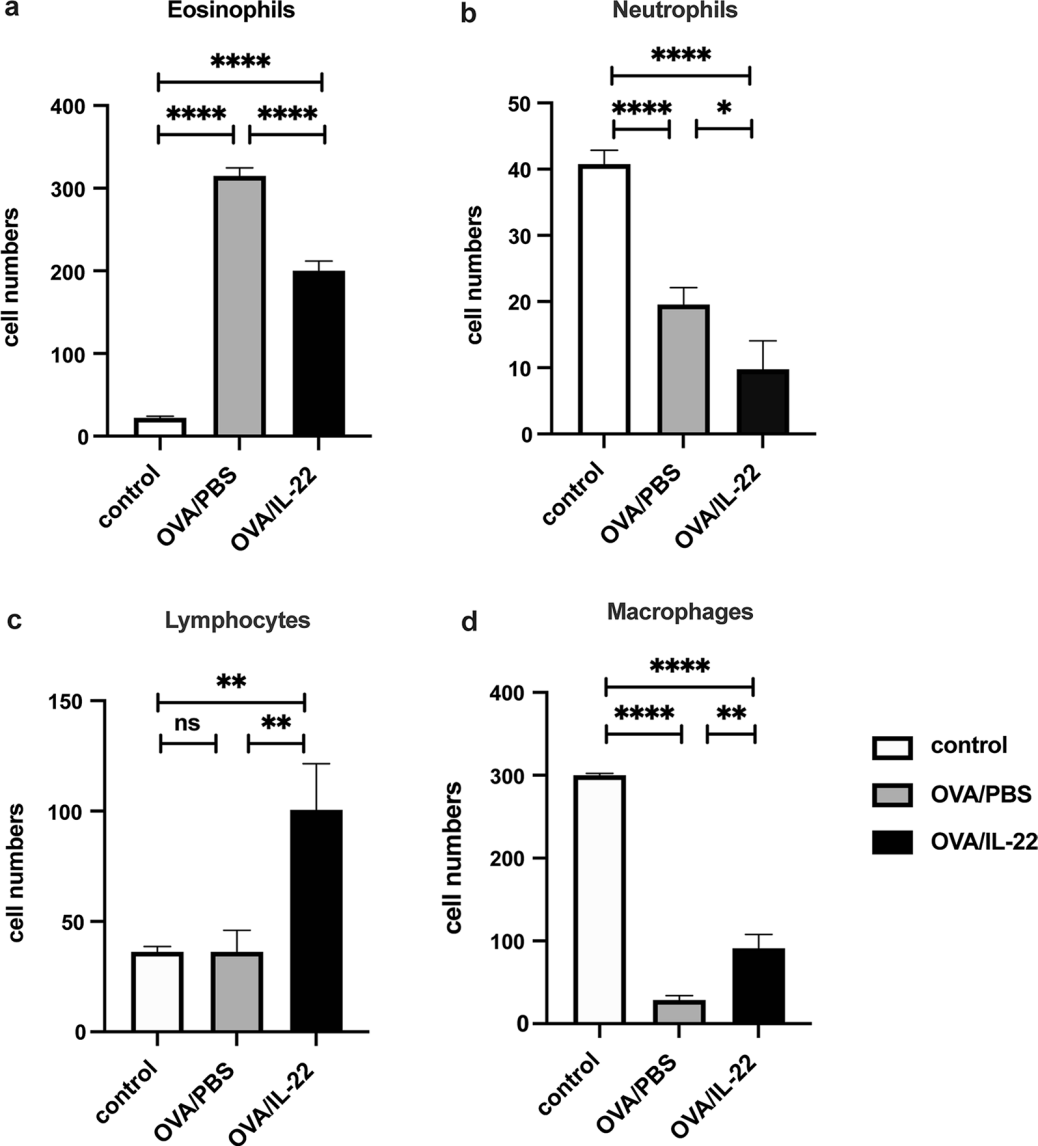
Intranasal administration of rmIL-22 attenuated OVA-induced eosinophil recruitment into the airways. Recombinant mouse IL-22 (0.2 µg per mouse) or PBS was given intranasally to OVA-induced animals three times in total at 24 h before the inhaled OVA challenge. Numbers of eosinophils (**a**), neutrophils (**b**), lymphocytes (**c**) and macrophages (**d**) in BALF at 24 h after OVA challenge. Bars are presented as the mean ± SEM (n = 5 in each group). * *p* < 0.05, ** *p* < 0.01, ** *p* < 0.001, **** *p* < 0.0001

Intranasal challenge with OVA was related with a significant elevation of the mean eosinophils infiltrating the peribronchial and perivascular regions of the lung tissues compared with PBS control, as shown by Wright-Giemsa staining, where 400 cells were counted in each section in continuous fields of vision; whereas intranasal administration of rmIL-22 dramatically decreased the mean eosinophils compare with the OVA-induced group (Fig. 3a, *p* < 0.0001), which was significantly, but not completely attenuated in OVA-induced mice compared with the control mice. We also observed a significant decrease in the mean number of neutrophils and macrophages in BALF of OVA-induced mice, compared with the control group; after IL-22 treatment, the mean number of neutrophils continued to decline compared with the OVA-induced mice (Fig. 3b, *p* < 0.05), while the mean number of lymphocytes and macrophages were significantly increased in the BALF of the IL-22 treatment group compared with the OVA-induced group (Fig. 3c and d, *p* < 0.01).

### IL-22 attenuated OVA-induced eosinophilic infiltration into lung tissue

**Fig. 4:**
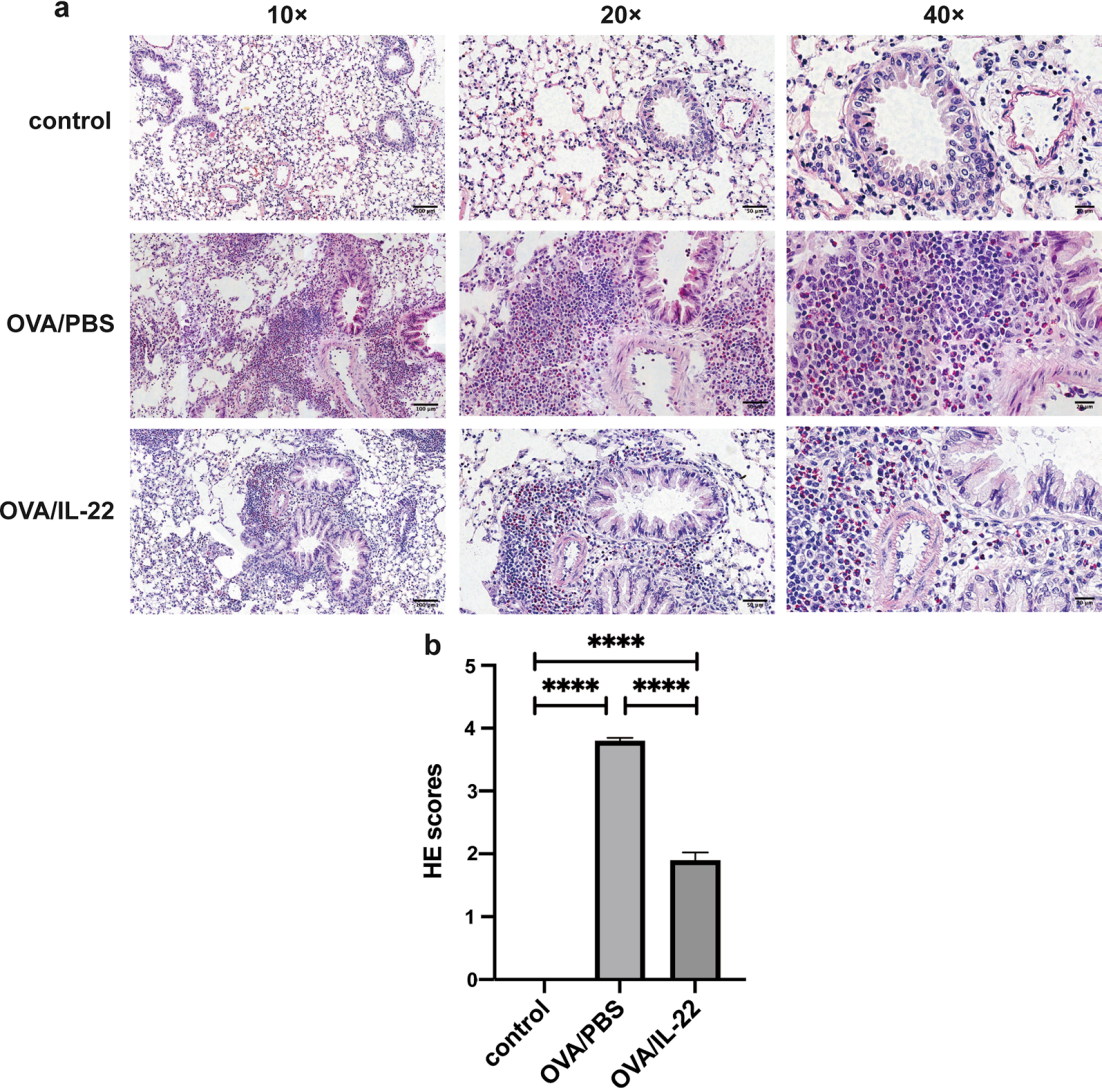
Effects of rmIL-22 on airway inflammation in OVA-induced asthma model. Representative images of lung tissues sections stained with H&E (**a**) (original magnification 10 × , 20 × and 40 ×), and the semi-quantitative analysis of the scores of inflammatory cell infiltration in the airways (**b**). All data are presented as the mean ± SEM (n = 8–10 in each group). ** *p* < 0.05, ** *p* < 0.01, ** *p* < 0.001, **** *p* < 0.0001

Intranasal challenge with OVA induced a significant increase in the total number of cells infiltrating the peribronchial and perivascular areas of the lung tissues, predominantly eosinophils, compared with control group (Fig. 4a). While H&E staining specimens of the rmIL-22 administration group revealed that the scores of peribronchial and perivascular inflammatory cell infiltration were significantly improved, compared with OVA-induced group (Fig. 4b), but not completely attenuated compared with control group (Fig. 4b).

### IL-22 influenced the receptor expression in lung tissues of OVA-induced mouse model

**Fig. 5:**
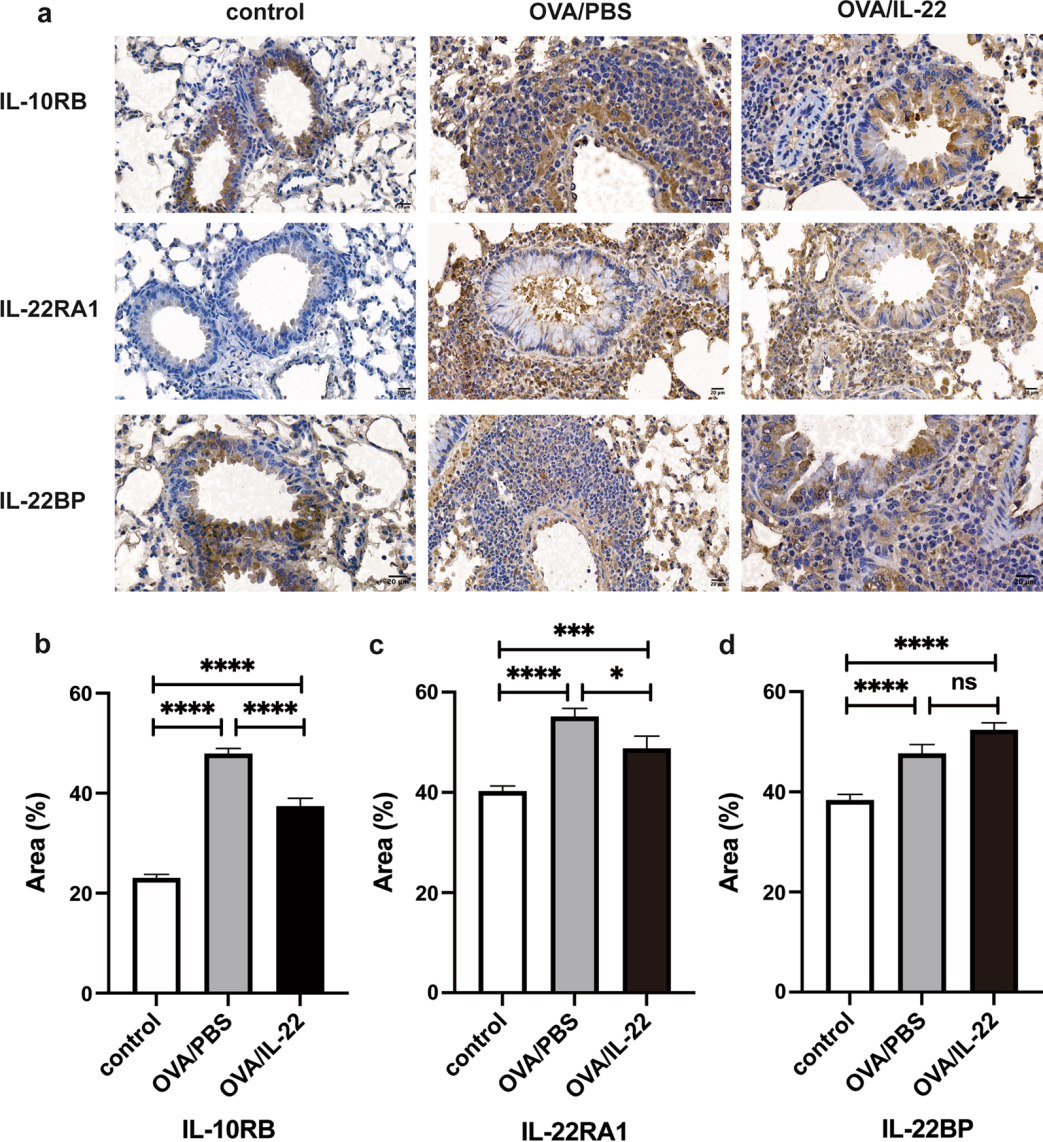
Influence of rmIL-22 on IL-22 receptors in OVA-challenged mice lung tissues by immunohistochemical staining. **a** Representative photomicrographs of IL-10RB, IL-22RA1 and IL-22BP immunoreactivity in sections of lung tissues in control, OVA/PBS, OVA/IL-22 group (original magnification 40 ×); **b**–**d**. The quantitative analysis of the expression of IL-10RB, IL-22RA1 and IL-22BP, respectively. Bars present the mean ± SEM (n = 3 in each group). **p < 0.05, **p < 0.01, **p < 0.001, ****p < 0.0001

To explore the potential mechanisms of IL-22 which inhibited the OVA-induced airway inflammation, we performed immunohistochemistry to detect the expression of functional IL-22 receptor, a heterodimer of IL-22RA1 and IL-10RB, and its soluble binding protein, IL-22BP. Immunohistochemistry analysis showed that the expression of IL-22RA1 and IL-10RB in the lung tissues of OVA-induced mice was significantly increased compared with the control mice (Fig. 5a–c, *p* < 0.0001), while was dramatically decreased after the treatment with IL-22 with statistical significance (Fig. 5a–c, *p* < 0.05), but was not completely attenuated in the IL-22-treated mice when compared with the control mice (Fig. 5a–c, *p* < 0.001). We also examined the IL-22BP expression which was significantly enhanced after OVA challenge (Fig. 5a and d, *p* < 0.0001), but no difference was noted after IL-22 administration (Fig. 5d, *p* > 0.05).

## Discussion

In this study, we have compared the expression level of IL-22 in OVA-induced mice and control mice, explored the effect of IL-22 on allergic asthma, and found that the IL-22 level significantly increased in lung tissues of the OVA-induced mice and IL-22 showed anti-inflammation properties in allergic asthma, attenuating airway inflammation and airway hyperresponsiveness.

Interleukin-22 is one of the lymphoid cell-derived cytokines, and have exclusive functions on non-hematopoietic cells, especially epithelial cells, playing a critical role in the regulation of allergic asthma airway inflammation [11, 31]. Previous studies have demonstrated that IL-22 level was higher in allergic asthma than in healthy control [19, 22, 32]. In our study, we detected the IL-22 level in lung homogenates and BALF in allergic asthma model mice and control mice. Consistent with previous findings, the expression level of IL-22 in allergic asthma mice was higher than that in control mice. This observation is firmly supported by that the majority of IL-22 is produced by CD4^+^ T cells in allergic airway inflammation [19]. This may suggest that IL-22 is involved in the pathogenesis of allergic asthma, and further studies are needed to confirm the precise roles of IL-22.

According to previous studies, IL-22 still exhibits controversial properties, both pro-inflammatory and anti-inflammatory, in allergic airway inflammation of murine studies [19, 20, 24, 33]. Previous findings have shown the anti-inflammatory roles of IL-22 in the allergic airway inflammation [19, 20, 34]. Ito et al. have researched that the house dust mite (HDM)-induced allergic airway inflammation and AHR are exacerbated in IL-22^−/−^ mice, indirect exhibiting the protective effect of IL-22 [20]. Fang et al. have generated IL-22 transgenic mice that overexpress IL-22 in the lung, which decreased eosinophils infiltration and reduced AHR in OVA-induced asthma model [34]. Takahashi et al. have adopted with anti-IL-22 antibody, neutralizing IL-22 and enhancing AHR [19]. In our study, we have administrated mice directly with the recombinant murine IL-22 in OVA-induced asthma model mice to investigate the role of IL-22, finding that allergic airway inflammation was significantly attenuated by IL-22 administration, including a decreased number of eosinophils in BALF, reduced inflammatory cell infiltration around the bronchi and their accompanying vessels and decreased AHR. Despite of existing some duplication of previous study, this study and previous studies complement each other and jointly illustrate the anti-inflammatory and protective effects of IL-22. These reasons may explain our findings: on the one hand, IL-22 gene delivery inhibited OVA-induced proliferation and cytokine production of CD4^+^ T cells and increased IL-10 production, an immunosuppressive cytokine [33]; on the other hand, due to predominant acting on lung epithelial cells, IL-22 inhibits the production of epithelial pro-inflammatory cytokines, IL-33, IL-25 and TSLP, by inducing the production of Reg3γ, an anti-microbial peptide, in allergic airway inflammation [19, 20]. However, in several previous studies, IL-22 has acted as a pathogenic effect on allergic airway inflammation [22–24, 35]. In skin inflammation and wound healing, IL-22 upregulates the proinflammatory gene expression in a dose-dependent manner, induces the keratinocyte migration and inhibits the epidermal differentiation [35]. In the fungi-induced allergic airway inflammation, IL-22 is indispensably required, IL-22 absence attenuates proallergic and proinflammatory responses, and neutralization of IL-22 improves lung function [23]; in addition, in epicutaneous-sensitized and OVA-induced allergic airway inflammation, the generation of CD4^+^ T cells producing IL-22 is promoted, meanwhile, promoting the production of IL-17A and exacerbating airway inflammation and AHR [24]. All above suggested that the protective and pathogenic roles of IL-22 may mainly depend on the routes of sensitization and the antigen species of sensitization and challenge.

Through the heterodimeric receptor consisted by the IL-10RB and the IL-22RA1, IL-22 mediated the downstream STAT3 activation in lung epithelial cells, improving epithelial repair and barrier function, and inducing the production of anti-microbial peptide [20, 36, 37]. IL-10RB is widely expressed on hematopoietic and non-hematopoietic cells, while IL-22RA1 is restrictedly expressed on non-hematopoietic cells, especially epithelial cells, determining the target cells of IL-22 [10, 11]. Previous study first reported that IL-22RA1 is expressed on the lung epithelial cells in OVA-induced airway inflammation [19]. Consistent with previous studies, we found that IL-10RB and IL-22RA1 were ubiquitously and restrictedly expressed, respectively; the expression of IL-10RB and IL-22RA1 significantly increased in OVA-induced mice lung tissues, and decreased after the administration of IL-22; but this recovery did not completely reach to the control mice level. Boniface et al. found that blocking IL-10RB and IL-22RA1 could affect the phosphorylation of STAT3, and demonstrated that IL-22 could induce STAT3 phosphorylation through IL-10RB and IL-22RA1 [35]. Therefore, it is suggested that IL-22 participates in the allergic airway inflammation by functional IL-22 receptor, IL-10RB and the IL-22RA1 complex. IL-22BP is a soluble binding protein of IL-22 and could inhibit the activity of IL-22, which was significantly increased in OVA-induced mice but no difference was found between the OVA-induced mice group and the IL-22 administration group in our study. IL-22BP gene expression was significantly decreased during influenza infection [38], and there was decreased inflammation, pulmonary injury and pulmonary edema in IL-22BP^−/−^ mice, compared with the wild type mice [39]. These suggest that IL-22BP plays a pro-inflammatory role, and further reveal the protective role of IL-22.

However, there are certain limitations in our study. First, we utilized the wild type mice but not the IL-22 knockout mice to clarify the protective effect on allergic airway inflammation, thus the influence of endogenous IL-22 could not be excluded. Second, we did not detect the effect on production of epithelial inflammatory cytokines. Finally, although we have detected the functional receptors of IL-22, the detailed downstream mechanisms of IL-22 in the OVA-induced asthma model should be further explored in the future studies.

## Conclusion

In summary, the present study clearly demonstrated the protective role of IL-22 in an OVA-induced asthma model. It also showed that IL-22 could suppress the inflammatory cell infiltration around bronchi and their concomitant vessels and airway hyperresponsiveness by its heterodimer receptors. Therefore, IL-22 administration might be an effective strategy to attenuate allergic airway inflammation.

## Data Availability

Upon request of corresponding author.

## Abbreviations

AHR: Airway hyperresponsiveness
ANOVA: One-way analysis of variance
BALF: Bronchoalveolar lavage fluid
CPH: China Pulmonary Health
Crs: Compliance
ELISA: Enzyme linked immunosorbent assay
Ers: Elastance
HDM: House dust mite
H&E: Hematoxylin and eosin
IFN: Interferon
IL: Interleukin
ILC3s: Group 3 innate lymphoid cells
IL-22BP: IL-22-binding protein
IL-22RA1: IL-22 receptor A1
Mch: Methacholine
OVA: Ovalbumin
PBMC: Peripheral blood mononuclear cells
PBS: Phosphate buffered saline
Rrs: Airways resistance
SEM: Standard error of mean
STAT: Signal transducer and activator of transcription
TSLPL: Thymic stromal lymphopoietin
WT: Wild-type
